# Was *Coccidioides* a Pre-Columbian Hitchhiker to Southcentral Washington?

**DOI:** 10.1128/mbio.00232-23

**Published:** 2023-03-07

**Authors:** David M. Engelthaler, James C. Chatters, Arturo Casadevall

**Affiliations:** a Translational Genomics Research Institute, Flagstaff, Arizona, USA; b Applied PaleoScience, Bothell, Washington, USA; c Johns Hopkins Bloomberg School of Public Health, Baltimore, Maryland, USA; Duke University Hospital

**Keywords:** *Coccidioides*, genomics, paleo-epidemiology

## Abstract

*Coccidioides immitis*, a pathogenic environmental fungus that causes Valley fever (coccidioidomycosis) primarily in the American Southwest and parts of Central and South America, has emerged over the past 12 years in the Columbia River Basin region, near the confluence with the Yakima River, in southcentral Washington state, USA. An initial autochthonous Washington human case was found in 2010, stemming from a wound derived from soil contamination during an all-terrain vehicle crash. Subsequent analysis identified multiple positive soil samples from the park where the crash occurred (near the Columbia River in Kennewick, WA), and from another riverside location several kilometers upstream from the park location. Intensified disease surveillance identified several more cases of coccidioidomycosis in the region that lacked any relevant travel history to known endemic locales. Genomic analysis of both patient and soil isolates from the Washington cases determined that all samples from the region are phylogenetically closely related. Given the genomic and the epidemiological link between case and environment, *C. immitis* was declared to be a newly endemic fungus in the region, spawning many questions as to the scope of its presence, the causes of its recent emergence, and what it predicts about the changing landscape of this disease. Here, we review this discovery through a paleo-epidemiological lens in the context of what is known about *C. immitis* biology and pathogenesis and propose a novel hypothesis for the cause of the emergence in southcentral Washington. We also try to place it in the context of our evolving understanding of this regionally specific pathogenic fungus.

## *COCCIDIOIDES* BACKGROUND

*Coccidioides immitis* and *Coccidioides posadasii* are the two known genetically and geographically distinct species of the dimorphic *Coccidioides* fungus, primarily found in the deserts of the American Southwest (1). *Coccidioides* has been considered a soil saprophyte that can articulate athroconidial spores that, once released into the air following soil disturbance, can infect a large diversity of mammals, including rodents, canids, and primates (both human and nonhuman) (2).

More recently, the *Coccidioides* parasite theory has been gaining traction, with genomic and biologic evidence suggesting that this fungus has evolved to feed on animal proteins (from carcasses) while in the saprophytic phase (3, 4). Comparative genomic analysis has demonstrated that *Coccidioides* has lost genes for plant protein breakdown and has acquired animal proteinases, including those to break down keratin (5). Additionally, *Coccidioides* has been associated with rodent and other mammal burrows under several studies leading to the small mammal endozoan hypothesis (3, 4); wherein *Coccidioides* infects small mammals and is maintained in an inactive spherule state in lung granulomas, which subsequently transform into spore-producing hyphae when the rodent or small mammal dies (from either disseminated disease or some other cause) (3). Lowered temperatures after death of the mammalian host allow the fungus to revert to the hyphal phase, and to grow and expand in the carcass. This endozoan hypothesis still posits a soil-based existence and is primarily limited to soils near burrows, middens, and buried infected carcasses, which have high organic material loads to support continued hyphal growth.

*Coccidioides* is only found in the Western Hemisphere, with the major region of endemicity in the desert Southwest of North America and locally hot arid regions in Central and South America (2). *C. immitis* is largely found in central California (CA) and occurs sporadically in southern CA and northwestern Mexico. *C. posadasii* is more hemispherically distributed: it is predominantly found in the Sonoran Desert of Arizona and northern Mexico, but is also endemic in southern Utah, New Mexico, Texas, northeastern Mexico, Guatemala, and several countries in South America (including Argentina, Brazil, and Venezuela) (2).

## THE WASHINGTON STATE *COCCIDIOIDES* EMERGENCE: THE PROBLEM

*Coccidioides* is an environmental fungus that is primarily localized to the North American desert Southwest and has not previously been documented in soils outside its hot and arid endemic zones. In 2010, an autochthonous case was reported in southcentral Washington (SC-WA); this apparent expansion in range has been suggested to be connected to global and regional climatic changes that would allow for increased environmental distribution and survival in a warming landscape. Whether a link to climate change is correct or not, it does not answer the “origin” question: from where, when, and how did the region become an endemic source of *Coccidioides*?

## HYPOTHESIS

We hypothesize that recently identified SC-WA *Coccidioides* cases are derived from local soil that had been contaminated by burial of an early/ancient diseased human or canine that was originally infected in the San Joaquin Valley region of CA and migrated or returned north to the Columbia River basin region, likely thousands of years ago.

## AN INTERDISCIPLINARY ASSESSMENT

Rather than assessing from a single perspective (e.g., climate change), it is critical to instead examine the hypothesis from an interdisciplinary view to identify the possible weaknesses that can and should be explored. As our hypothesis describes the possibility of a single emergence-driving event thousands of years ago, it is not readily empirically testable and, therefore, should be assessed based on the confluence of biological, geographic, climatic, and archaeological lines of evidence, including, our understanding of the epidemiology of the pathogen and its disease, the ancient natural history of hosts, the paleo-climatic and geologic history of the locale, and the use of genomic data to infer the evolutionary history of this unique population of *Coccidioides*.

**FIG 1:**
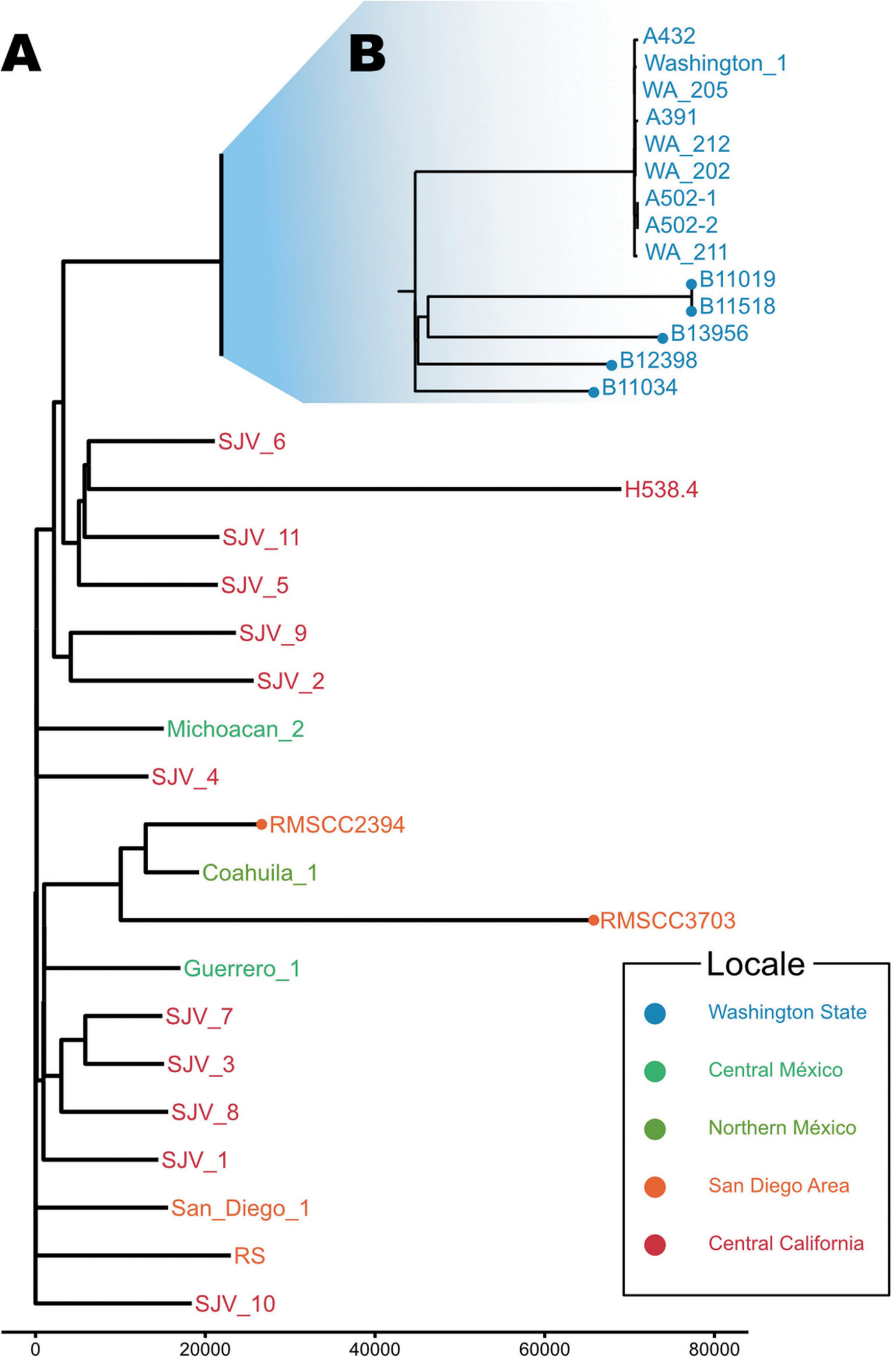
Maximum parsimony whole genome phylogenies of (A) *Coccidioides immitis* and (B) *C. immitis* strains from South Central WA. SNP distances are represented on numbered line. Adapted from Oltean et al. 2019 (6) and Monroy-Nieto et al. 2023 (9).

**Genomic evidence.** The 12 published genomes from the SC-WA *Coccidioides* case and soil isolates make up a distinct phylogenetic clade of *C. immitis* (6). This SC-WA clade of *Coccidioides* is highly clonal compared to the larger population structure of *C. immitis* (6–9) (Fig. 1). Only one mating type has been found among the WA genomes (8) (a likely reason for the limited population diversity observed), which can also be an indicator of limited or single introduction of a common ancestor in the region. This clade does, however, contain hundreds of single nucleotide polymorphisms (SNPs), suggesting sustained local replication, evolution, and diversification. Using the coarse 10^−9^ substitutions/year estimate previously published for *Coccidioides* and an ~29 Mb genome (8), the published 234 to 324 SNP mutation distance between individual genomes in the SC-WA region (6) could be grossly estimated to represent about 7 to 10K years of evolution. Although not suspected here, if we assume the much faster mutation rate of 10^−8^ substitutions/year rate, as has been seen in recombining fungal populations, the calculation yields a most recent common ancestor at 700 to 1,000 years ago. Additional, more refined, molecular clock estimations (e.g., employing Bayesian evolutionary inference tools) will add better confidence to age of this population. In either situation, however, these calculations establish a Pre-Columbian time frame for introduction to the region.

As a whole, *C. immitis* is an old species, likely separating from *C. posadasii* at least five million years ago (mya) (5), and further diversifying approximately one mya in what is now the San Joaquin Valley (SJV) in central CA (8), a large former inlet of the Pacific Ocean that currently houses one of the most productive agricultural lands in the world. The SJV is the major endemic source of *C. immitis* and provides the disease’s colloquial namesake “Valley fever.” The SC-WA genomic clade fits within a larger SJV-CA population of *Coccidioides*, suggesting a possible originating locale (6) (Fig. 1). While population studies of *C. immitis* are still limited, it is surmised that the SJV-CA population is the oldest and most diverse phylogeographic group of *C. immitis*, consisting of a number of local subpopulations and other endemic locales that have distinctly diverged local phylogeographic clades (e.g., southwest CA and Baja Mexico) (8). The population structure, therefore, signals both temporal and geographic origins of the SC-WA clade.

**Alternate interpretations of direction and timing of spread.** As the hypothesis proposes not only a mechanism (infected host burial) but also proposes directionality (CA to WA) and timing (several thousand years ago), it is important to consider the possibility of the contra-hypotheses: (i) not CA to WA, and (ii) not ancient. We briefly consider those now.

**(i) Directionality.** We identify three possibilities of dispersal direction of *C. immitis* associated with WA: (i) SJV-CA to SC-WA, (ii) SC-WA to SJV-CA, and (iii) a third locale dispersing to both SJV-CA and SC-WA. The primary evidence for Possibility 1 is the phylogenomic data that establishes the SC-WA population as a single clone that is distal, rather than basal, in the *C. immitis* phylogeny, which would negate possibility 2 (WA to CA) but not possibility 3 (third locale). Possibility 3 is not completely discordant with the phylogenomic data, as a separate originating source could have fed both the CA and WA locales independently. However, the SJV-CA populations are clearly more diverse and older than any other known *C. immitis* populations, and possibility 3 would require an even older and likely more diverse *C. immitis* population that is either undiscovered or is now extinct. Given the proliferation of genome sequencing on strains that have been recovered in most locales where coccidioidomycosis has been found, it is unlikely that there is a surviving diverse progenitor *C. immitis* population; however, the possibility for an extinct founding population that sourced both SJV-CA and SC-WA independently cannot be ruled out on genetic evidence alone. In that case, such a progenitor would have to: (i) have become extinct following dispersal to SC-WA, and (ii) would have had to disperse to SJV-CA and SC-WA at dramatically different time points and to nowhere else that has been detected to date (otherwise such a tertiary dispersed population would show as a basal in the *C. immitis* phylogeny).

**(ii) Timing.** As with directionality, there are also three possibilities for the timing of dispersal: (i) the SC-WA population is younger than the SJV-CA population, (ii) the SC-WA is older than the SJV-CA population, and (iii) the SC-WA and SJV-CA are the same age. We argue for the likelihood of possibility 1, given that genomic analysis of SC-WA indicates that the limited diversity results from a shorter evolutionary history. Possibilities 2 and 3 could only be possible if the SC-WA population has been in a suspended state of evolution, which could occur if isolates inhabited frozen environments. This concept is not without precedent: We have previously proposed that following the speciation event that separated *C. immitis* from *C. posadasii*, the former may have been held in glacial refugia in the Sierra Nevada glaciers prior to drainage of the central basin of California ~700 kya, which could account for millions of years of timing separation between observed initial diversification events in each species (8). This hypothetical mechanism has not been proven and requires further analysis; however, at this point, the most parsimonious answer is that the SC-WA population is evolutionarily newer than the SJV-CA population.

Given the several possibilities for the origin of the SC-WA isolates with regard to directionality and timing, we now further explore biogeographic, host/vector, and soil-contamination lines of evidence to identify the most likely originating scenarios.

**Biogeographic evidence.** Currently, the only known endemic soils for *C. immitis* in the Pacific Northwest (PNW) region are the soils that have been found in the Tri-Counties region of SC-WA over the past decade. The first two human cases were linked to contaminated soils found along the Columbia River at the site of an accident at an ATV park, and to positive soil sites upstream near the Yakima River confluence (aka “the Delta”) with the Columbia River (7, 10). Multiple other cases with known exposure to the region have been since identified, and all genomes sequenced from local cases and soil isolates belong to a single SC-WA clade (6). Numerous other PNW coccidioidomycosis cases in WA and neighboring OR have been identified during this time period, but none have been linked to local contaminated soils, and most had travel history to known regions of endemicity during their likely exposure period (6, 9). One possible exception is a case in Spokane, WA (~210 km northeast of the endemic locale), which reported travel exposure to the SC-WA region >8 years prior to onset of disease (11).

**FIG 2:**
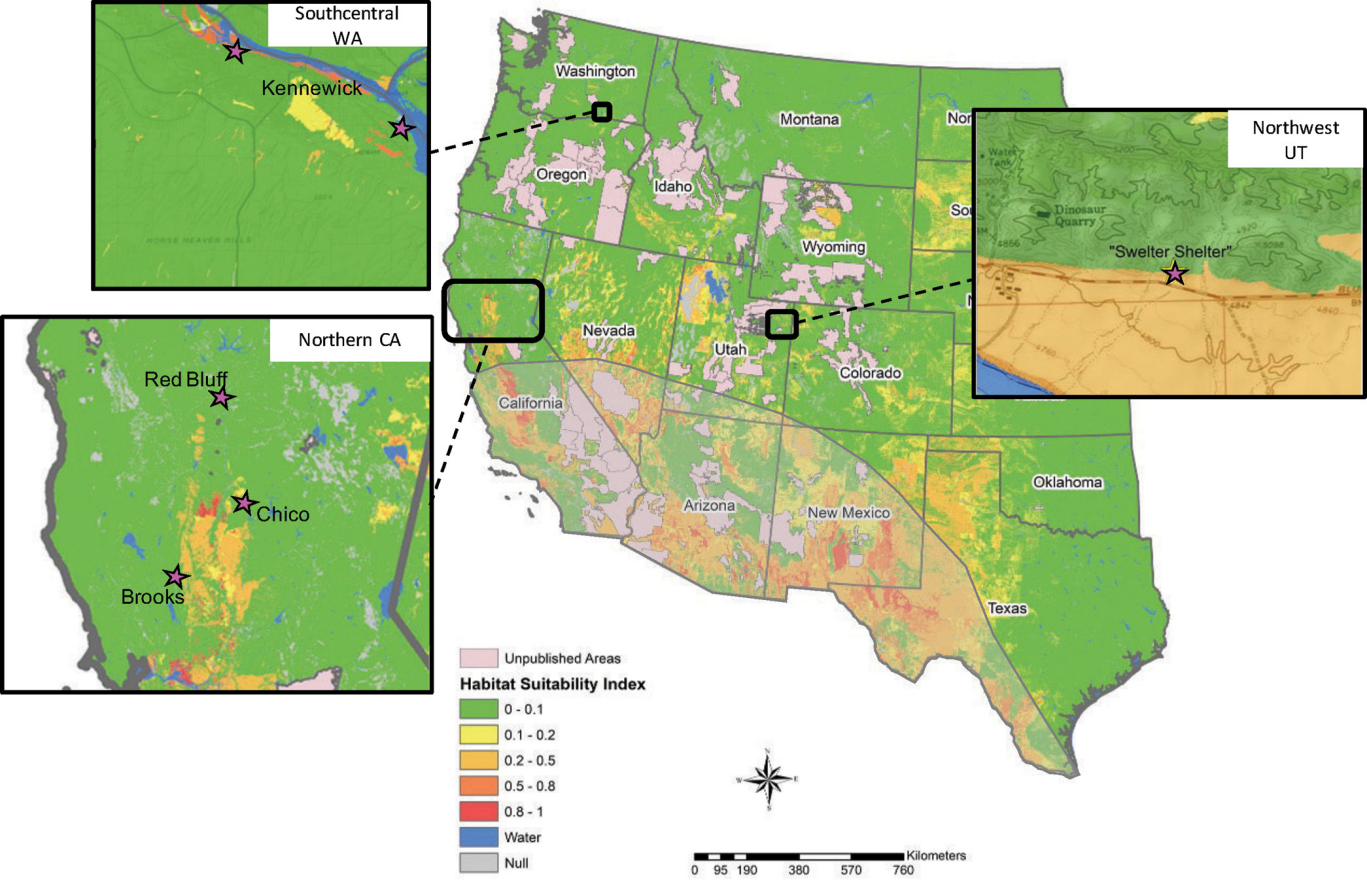
*Coccidioides* spp. soil habitat suitability in United States, including outbreak locations outside the classic endemic zone. Stars represent locales of archeological outbreak sites in California (CA) and Utah (UT), and locales of positive soils in southcentral Washington (WA). Lightly shaded area represents classic endemic zone. Adapted from Dobos et al. 2021 (15).

Endemic *Coccidioides* locales are typically limited to dryer, thermic soils in the Western Hemisphere, and notably include the SJV and the Sonoran Desert, where most human cases occur. Other endemic locales in the Southwestern US, Mexico, Central America, and South America are also all associated with arid locations with thermic soils (12). The SC-WA Tri-Cities region is a warmer, drier environment than much of the surrounding region and therefore appears to be a suitable habitat niche for the fungus (13, 14). A recent high-resolution habitat niche modeling study based on multiple soil and climate parameters (15) determined that the SC-WA locale was highly suitable for *Coccidioides*. This included nearby areas immediately adjacent to the Yakima River, which feeds into the Columbia River (Fig. 2), as well as limited locales in nearby northcentral Oregon (OR); although there is no current evidence of *Coccidioides* existing naturally in OR soils. The same study found that most of the surrounding area in WA and OR is considered unsuitable habitat (15).

**Animal infection evidence.** As the connection to mammals is a critical part of the *Coccidioides* life cycle and, as proposed here and elsewhere, to the distribution of *Coccidioides* into previously nonregions of endemicity (8, 16), one should consider the likelihood of the emergence in SC-WA being tied to an infected human or animal being buried or deposited in the soils. Local mammalian wildlife and positive soil samples have been identified at nearly all established endemic locales, which provides evidence of natural circulation (17).

*Coccidioides* is typically found in soil near small mammal burrows, and its parasitic life cycle has long been tied to mammal infection (3, 4, 18, 19). Genomic studies have provided significant evidence of the critical nature of animal infection for *Coccidioides* metabolism and survival, especially with genomic identification of decreases in plant proteinases and increases in animal proteinases (5). Previous studies have also established that *Coccidioides* presence in the soil tends to remain very focal, with nearby soil (<10 m away) often remaining uninfected (20); this finding would not be seen if distribution was either random or more ubiquitous in regions of endemicity, as would occur with a pure soil saprophyte. A previous study demonstrated that uncontaminated soil in the area of endemicity of *C. immitis*, which had failed to produce cultures of the fungus for 3 years prior to the experiment, became *Coccidioides*-positive 5 months after the burial of experimentally infected mice; this soil remained positive for each of the subsequent 6 years (21). Consequently, there is continued and growing support for the hypothesis that contaminated soils originate from an infected animal corpse (4) which, if the soil and niche conditions are suitable, results in long-term or permanent colonization of nearby soils (22). Both animal burrows and surrounding soils have been found positive in the SC-WA locale, although more study is needed to understand the extent of the burrow-surrounding soil relationship in the region (22).

**Evidence for single-introduction event.** It is reasonable that distinct phylogeographic populations result from discrete microbial dispersal events (e.g., specific one-time migration of, and subsequent site contamination by, infected animals; or significant landscape-changing events such as natural disasters) versus ongoing mechanisms of continually repeated events (e.g., seasonal animal migrations; or wind dispersal); otherwise, phylogenetic populations would lack geographical distinction (8). Based on its discrete phylogeographic population structure, *Coccidioides* likely dispersed to new endemic zones in Central and South America by the movement of infected mammals that subsequently died and decayed in the soil. In this regard, the timing of the dispersal from southwestern North America to South America coincided with the later Great American Biotic Interchange movements between continents more than 700 thousand years ago (kya) (8, 16), well before human presence in the hemisphere. Similarly, the clonal *C. immitis* population in SC-WA is likely to represent a single introduction event since multiple introductions from a presumable SJV-CA source locale would have resulted in a polyphyletic distribution with a common ancestor shared between isolates originating elsewhere. A similar effect could have been responsible for the recently investigated clonal Venezuelan *C. posadasii* population (23), which may also have been the result of an introduction of a single *C. posadasii* infected animal to the locally restricted region of endemicity.

**Evidence for soil contamination through human and companion animal burial.** Beyond infected wildlife and before the modern era of burial practices that would prevent soil contamination from infecting fungi, humans and their canine companions also likely made for suitable vectors to translocate fungus to new regions. *Coccidioides* has been found in multiple archeological sites where human and animal (canine) bodies have been buried; a famous nonendemic *Coccidioides* hot spot (i.e., highly localized soil presence and documented human cases) is the Swelter Shelter archeological dig site at Dinosaur National Monument in northern UT (12) (Fig. 2). The immediate region has been identified as a moderately suitable habitat niche (15), and although this locale in northern UT is well outside the standard endemic zone, it is frequently included in region of endemicity maps due to documented outbreaks occurring at the Swelter Shelter site. Archeologist outbreaks in both 1964 and 2001 (24–26) were directly linked to exposure to a single highly contaminated Swelter Shelter midden that was subsequently shown to be *Coccidioides* PCR-positive (27). These outbreaks had extremely high attack rates (>90% of susceptible individuals were infected) (26) owing to the highly localized and intense nature of exposure to the contaminated site. This conclusion is further supported by a lack of positive case finding or serologic evidence of exposure in longtime workers at Dinosaur National Monument who did not have direct exposure to the Swelter Shelter site (26).

Previous studies have identified bone lesions consistent with *Coccidioides* in human skeletal remains from pre-Columbian burial sites in highly endemic Arizona regions (28, 29). However, given the lack of any known human grave at Swelter Shelter, it is possible that the Swelter Shelter *Coccidioides* contamination is from the burial of pre-Columbian companion canines, which could have been infected with *Coccidioides* elsewhere and subsequently died and buried in the midden, where the fungus was able to survive and thrive within the protected site. Buried animal skeletal remains at Swelter Shelter were found in the deepest, oldest layer in the midden, dating back to nearly nine thousand years ago (24).

Similar coccidioidomycosis outbreaks have been described at other archeological sites in the Western US, including multiple locales outside classic *Coccidioides* areas of endemicity (Fig. 2). A 1968 coccidioidomycosis outbreak occurred in archeologists at a dig site in Brooks, CA (40 miles north of San Francisco), well outside the classic endemic zone (30). Additionally, a large 1970 outbreak involved 61 archeological students at a dig site with human remains and middens near Chico, CA (100 miles north of Sacramento) (31). In 1973, an outbreak of 17 archeology students occurred at a site of ancient Yana people in Red Bluff, CA (20 miles north of Chico, CA) (32) (Fig. 2). Each of these instances had high attack rates (59%, 44%, and 48%, respectively), providing more evidence that archeological sites in the American West are at high risk for being heavily contaminated *Coccidioides* locales, even if outside the region of endemicity. Perhaps less significantly, a fossilized ancient *Bison antiquus* jaw bone, recovered in Nebraska and dating to ~8,500 years ago, was thought to contain lesions that were indicative of possible *Coccidioides* spherules (33). Subsequent ancient DNA analyses of bone material collected from the lesion did not detect coccidioidal DNA but did successfully amplify bison DNA (D.M. Engelthaler et al., unpublished data), leaving a hypothetical ancient *Coccidioides* presence in Nebraska as still unsupported.

**A human or canine burial site connection to the southcentral WA locale?** The region of the SC-WA *Coccidioides* population centers around Kennewick, WA (Fig. 2)—the location of the discovery of “Kennewick Man,” or “The Ancient One”—from approximately 9,000 years ago (34–36). This coincidence is interesting because he was either purposely buried at the site or covered by flood sediment soon after death, making him the earliest known interment in the region. This is not to suggest that The Ancient One was tied to the local *Coccidioides* population in the region; rather, we mention Kennewick Man because he demonstrates the occurrence of ancient burials in the area, which supports the notion of local soil contamination via burial. In fact, human bodies were historically buried throughout the southern Columbia Basin, with some of the earliest recovered from cave and rockshelter sites in deposits almost as old as Kennewick Man (37). Earlier interments (5,000 to 9,000 years ago) have been identified at Marmes Rockshelter, ~100km northeast of Kennewick, while a grave was identified at Cedar Cave, on the Columbia River above Kennewick, dating to approximately 2,500 years ago. Open air interments were common on the Columbia River dating from at least 3,000 years ago (37), including islands at the nearby mouth of the Yakima (37) and those at the Wahluke Site, located further upstream of the Yakima River (38). Interestingly, one human skeleton, uncovered in the 1987 Hanford site (along the Columbia River), dating to 1800 years before present, had bone lesions that were originally thought to be associated with Brucella, but are also consistent with a possible *Coccidioides* myelitis (39).

Dog burial was practiced by people living a short distance downstream from the SC-WA area of endemicity as early as 2,500 years ago (40). In addition, small canids, probably dogs, were a common food element along the Columbia River and are incorporated into archaeological middens dating between about 9,000 to 5,000 years ago (41, 42). It is clear that humans and other animals have been buried in the region and that such burials can result in soil contamination, possibly with pathogenic organisms not endemic to the region.

**Ancient and modern climate change.** The climate conditions in western North America were significantly different >4,000 years ago than they are today. The region north of the Columbia Basin was glaciated episodically during the Pleistocene Epoch, which had its most recent glacial maximum >19 kya. Subsequent warming (interrupted briefly by glacial readvance known as the Younger Dryas) resulted in the elimination of large glaciers in the region by less than 12 kya. Although offset by colder winters, rapid warming continued, raising regional summer temperatures to levels more than 1.2°C above the modern average by ~9,500 to 9,000 years ago (43). The period between 9,500 and 6,400 years ago was the most arid for the Columbia Basin, with hotter summers (reaching ~1.8°C% above the modern summer average temperature of 27.3°C) paired with warmer winters. Vegetation patterns also indicate lower regional precipitation (~14cm) in the arid Kennewick area during this time point (modern Kennewick area annual precipitation is ~19.5 cm). These conditions may have promoted initial *Coccidioides* survival and growth in the immediate area of where an infected body may have been buried (36). In fact, the increase in warming and aridity is likely to have had the effect of expanding the suitable habitat of *Coccidioides* in the Columbia Basin beyond its current known range, which occurred during a time when people were interring their dead in primary graves. Climate became gradually moister and cooler after 6,400 years ago, with readvances of montane glaciers occurring episodically after 5,000 years ago. This cooling increased between 4500 and 4000 years ago and included two widespread episodes of markedly cooler, moister conditions. The first, known as the Neoglacial, lasted from ~4,000 to 2,800 years ago, while the last, known as the Little Ice Age, persisted from 400 years ago until the late 19th century. While ancient climate change may have provided the conditions for *Coccidioides* to initially survive and thrive in the soils in the region ahead of the significant cooling of the “mini-ice ages,” warming since this glacial period may have provided an impetus for continued soil growth, *Coccidioides* cycling in small mammal populations, and subsequent local transmission.

**Translocation to the Pacific Northwest.** This then begs the question of how likely was it that a local resident (human or canid), thousands of years before regular long-distance travel, could have been infected with *Coccidioides* in central California and traveled hundreds of kilometers north to eventually die, be buried, and contaminate soils with a nonendemic fungus. We obviously do not have records of such travels, but we can look to seashells for a clue. We know *Olivella* shell beads from the nearby Fort Rock Basin of central Oregon, dating to the middle and late Holocene (<9,000 years ago), came from the lower Pacific coasts (44) and that trade in that comodity existed at the same time in eastern WA (45). *Olivella* beads were found associated with most of the early human burials at the nearby Marmes Rockshelter, including two from a stratum dating >7,500 years (46). Further, it is well documented that there were encounters and trade between peoples from central CA valley and coastal CA for most of the last 10K years (47); ancient trade routes throughout North America allow for transcontinental travel of material passing through the hands of traders that individually travel shorter distances. Beyond trade routes, the Columbia River salmon runs were a great annual draw for outside populations, which led to ephemeral seasonal migration to the region (48); and climatic conditions for massive salmon runs have persisted for the last 4,000 years (49). Additionally, it is possible that other long-distance travel for resettlements, nomadic lifestyles, or ritual quests occurred that are not otherwise documented in historical record over the millennia.

We know that domesticated canids (Canis familiaris)—which are highly susceptible to *Coccidioides* infections—are frequently found in ancient burial sites and middens in the region, which stands to reason, given the close historical relationship between humans and dogs, as pets, protection and providers of fur and food (50). Dogs were also valuable trade items (50) and would have certainly been made to travel long distances as they were passed between traders. Wild canids (wolves and coyotes) were also present, and they too travel long distances; current knowledge suggests far-reaching wolf (*Canis lupus*) territorial hunting grounds (up to tens of thousands of square kilometers) (51) and natal dispersal of over 3000 km (52), with coyotes (*Canis latrans*) having much smaller territories (140 km^2^) and dispersal distances (160 km) (53). However, such wild canids are rarely found in burial sites or middens (50), and carcasses of dead wild animals would likely have been scavenged rather than causing significant contamination below the topsoil.

There is also the possibility of other infected mammals having been translocated or migrated from central CA to the Columbia River basin; however, most mammals are not known to be as susceptible to long-term or systemic infections as humans and canines. Although a number of mammal species have been identified as having been infected with *Coccidioides* (54), aside from wild felids and rodents (which do not have large territories or long-distance dispersal), none are known to have existed in the ancient Columbia River basin. An interesting finding of *Coccidioides*-positive bats in Brazil (55) and positive bat guano in caves in southern AZ (56) has led to another untested long-distance dispersal hypothesis via bat migration, which could then occur over thousands of kilometers.

In summary, the evidence as presented above is consistent with the hypothesis of ancient *Coccidoides* dispersal from CA to SC-WA: (i) endemic *Coccidioides* from SC-WA is genetically distinct from that found in the southern Central Valley of CA, with divergence times as early as 7000 to 10,000 years ago and as late as 700 to 1000 years ago; (ii) human burial was practiced since at least 9000 years ago and dog deposition in middens goes back nearly as long (dog interment was practiced between ~2500 and 1000 years ago); (iii) continental trade routes, in the form of marine shells from the coast of southern CA to SC-WA, is known from as early as the middle Holocene; and (iv) climatic conditions during the mid-Holocene, especially in the period between 9000 and 6000 years ago, were even warmer and drier than at present, providing suitable habitat for *Coccidioides*.

**Climate and other possible hypotheses for translocation of *Coccidioides*.** While postindustrial climate change has been recently invoked as a mechanism for the expansion of *Coccidioides* into previously nonregions of endemicity (13, 57), as a hypothesis it lacks an introduction event. In this regard, *Coccidioides* is clearly not instantaneously appearing in regions where the climate has become warmer and/or drier. Most habitat niche modeling primarily focuses on climatic variables and vegetation cover (58, 59), and therefore changes in such variables (e.g., warming climate and floral displacement) lead to predictions of changing endemic zones (13). However, if climate and its effects on soil alter local conditions, it is possible that either an earlier or subsequent soil introduction event—such as a buried infected carcass—may allow for the “emergence” of a new highly focal endemic locale.

Soil biochemical and microbial properties may be equally important. Biochemical properties of *Coccidioides*-positive soils versus -negative soils have been studied (4, 20, 22), and although differences have been found (e.g., elevated concentrations of boron, calcium, magnesium, sodium, and silicon in soil leachates were documented in WA-positive versus -negative soils) (22), it is thought that *Coccidioides* may likely survive in most native soils found in the regions of endemicity with local rodent populations (58, 60). It has also been demonstrated that at least some soils in the American Southeast are not suitable for *Coccidioides* maintenance and growth (22). It is not clear that climate patterns can change the biochemical properties of the soil that would make once inhospitable soil conducive to coccidioidal growth. The lack of a permissive environment may actually be due to the combination of climate, biochemical properties, and the presence of microbial agonists (22, 61); for example, for other soil fungi such as Cryptococcus neoformans and *Paracoccidioides* spp., soil residence is directly affected by the presence of amoebae (62, 63). While the interaction between *Coccidioides* spp. and amoebae has not yet been studied, it is possible that microbial factors are also important for its success in contaminating certain locales. Therefore, future modeling of specific microbial habitat niche may need to employ multiple factors, including soil composition, moisture, temperature, and both vegetative and microbiota composition.

A recent such model pinpoints the areas where *Coccidioides* is known to be endemic—including the specific SC-WA locales where *Coccidioides* has been detected in the soil—as having moderate to high habitat suitability (15). This has also been found in areas near the northern CA outbreak sites but identifies the Swelter Shelter site in UT as having only moderate habitat suitability (Fig. 2). This is likely because the positive midden sediments at Swelter Shelter are protected and were probably highly contaminated with an infected carcass. The model proposed by Dobos et al. (15) also pinpoints small suitable locales in Nebraska, Idaho, Wyoming, and South Dakota, where locally acquired coccidioidomycosis or positive soils have not yet been identified. It may be that the limiting factor is not soil conditions or climate, but, rather, whether an infected animal, human or otherwise, died in a region that either has highly or at least moderately suitable soil and is also protected from unsuitable climate conditions (e.g., archeological sites or middens) in the American West. As such, any archeological excavation projects conducted in these regions, particularly if the sites suspected to include human or canine remains, should maintain the same coccidioidal prevention precautions as are currently used in known regions of endemicity (26).

An alternate dispersal hypothesis is that *Coccidioides* is translocated by wind storms and other natural disasters (64). This is typified by the infamous case of distant coccidioidomycosis cases associated with a windstorm natural disaster event: the 1977 “Tempest of Tehachapi” (65). This massive windstorm carried >10 cm of topsoil westward over vast distances and clearly resulted in airborne transport of *Coccidioides* spores from SJV-CA, resulting in coccidioidomycosis cases as far away as San Francisco and Sacramento (hundreds of kilometers) (65). Additionally, the 1994 Northridge, CA, earthquake caused a significant outbreak over a large distance due to large amounts of soil and spores being aerosolized (66); however, neither of these disaster events is known to have resulted in newly regions of endemicity. That is, airborne coccidioidal fungal spores can travel great distances and end up causing cases in nonregions of endemicity; however, spores that settle onto the top layers of soil are unlikely to result in new local endemic foci. This is probably best evidenced by the fact that we can see distinct *Coccidioides* population structures between endemic locales that are connected by winds (e.g., Phoenix and Tucson, AZ), whereas windblown translocation would result in indistinguishable heterogeneous populations (8). Interestingly, while dust storms would seem to be an obvious source of *Coccidioides* exposure (67), air monitoring during a documented massive dust storm (aka “haboob”) in the highly endemic metropolitan Phoenix region did not result in an increase in detectable airborne fungal spores (68), and epidemiological association studies have not identified a clear link between documented dust storms and increased human cases of coccidioidomycosis (69). None of these arguments discount the likely and obvious connection of local epidemiology to climate, which has been explored and modeled for at least 2 decades (14, 59, 70–72). It is generally understood that temperature and rainfall patterns combine to provide suitable soil conditions for fungal mycelial growth, which leads to spores becoming airborne following soil disturbances (aka “grow and blow”) (71). However, this environmentally driven coccidioidomycosis epidemiology does not have a mechanism for habitat dispersal beyond this natural cycling resulting in the infection of a susceptible mammal that can travel long distances and die (and perhaps be buried) elsewhere, with the resulting carcass heavily seeding the soil with a novel microbe.

**Context with modern climate change.** Currently, nearly all discussions of environmental pathogen dispersal and expanding zones of endemicity seem to be centered on contemporary global warming trends. While climate change may in fact be playing an important role in creating both subtle and overt changes to different ecological zones and microhabitats, we must account for how, when, where, and why microbes, such as fungi, disperse from one region to another. Climate change may then help provide ripe conditions for growth of recent or ancient deposition of environmental microbes. Additionally, if such a microbe is associated with ancient peoples in a region, this may represent a risk factor for future archeological exploration in said region. Climate change, therefore, may not always be a driver of pathogen expansion; however, it may reveal past such events in a deadly fashion.

## CONCLUSIONS

Reconstructing events in the distant past is important to understand the epidemiology of infectious diseases today; however, all such explorations are limited by the fragmentary information and the many possibilities that follow. To explain the *Coccidioides* presence in SC-WA, we have attempted to reconstruct the events leading to the introduction of this fungus to that site by piecing together genomic, anthropological, and climatic evidence with what is known about the biology and pathogenesis of coccidiomycosis from observational and experimental studies. Using Occam’s razor’s dictum that the simplest explanation is more likely to be correct, we propose that the SC-WA soil was contaminated via the travel, death and burial of an ancient human or domesticated animal from the central CA region of endemicity to SC-WA. Recent large-scale analyses of clinical cases have established the occurrence of human coccidiomycosis well outside known regions of endemicity (9, 73) and, while nearly all such occurrences are likely travel-related, it is possible that other locales contaminated with *Coccidioides* will be identified. Should those sites be found, the application of the same type of analysis as described here may further shed light on the mechanism by which *Coccidioides* can spread from its ancestral southwestern sites to colonize other regions.
